# miRNA in Plasma Exosome is Stable under Different Storage Conditions

**DOI:** 10.3390/molecules19021568

**Published:** 2014-01-27

**Authors:** Qinyu Ge, Youxia Zhou, Jiafeng Lu, Yunfei Bai, Xueying Xie, Zuhong Lu

**Affiliations:** 1 Key Laboratory for Child Development and Learning Science, Ministry of Education, Southeast University, Nanjing 210096, China; E-Mails: zhouyouxia1021@163.com (Y.Z.); xxying99@hotmail.com (X.X); 2 State Key Laboratory of Bioelectronics, Southeast University, Nanjing 210096, China; E-Mails: lujiafeng1984@163.com (J.L.); whitecf@seu.edu.cn (Y.B.)

**Keywords:** exosome miRNA, storage condition, stability

## Abstract

Exosomes are small membrane-bound vesicles secreted by most cell types. Exosomes contain various functional proteins, mRNAs and microRNAs (miRNAs) that could be used for diagnostic and therapeutic purposes. How we should store the samples before RNA isolation and whether those long term stored samples could be used for circulating RNA investigation because of RNase is unknown. The aim of the study was to determine the stability of circulating miRNA in exosomes and plasma. Exosomes were isolated from plasma samples by using ExoQuick Precipitation methods. RNA was extracted from exosomes and the corresponding plasma samples with a Qiagen miRNeasy Mini kit. The concentration of RNA was measured by a Qubit^®^ RNA HS Assay Kit, and quantitative PCR was used for individual miRNA expression level detection. Results showed that exosomal miRNA showed extra stability under different storage conditions and no significant influence on plasma miRNA, except for short term storage at 4 °C. It is thus indicated that exosome miRNAs can be good biomarkers based on their stability under various storage conditions.

## 1. Introduction

Cells release various types of membrane vesicles into the extracellular space that differ in origin, size, morphology and content [1]. The most studied of them are exosomes, small (40–100 nm) vesicles of endocytic origin. Exosomes can be easily purified from cell cultures and a number of patient body fluids, including plasma/serum, saliva and urine, *etc.* [2]. These extracellular vesicles contain various functional proteins, microRNAs (miRNAs) and mRNAs [3] which have the potential to be used as biomarkers for numerous diseases.

MicroRNAs (miRNAs) are common 19–22 nucleotide long, non-coding RNA molecules that post-transcriptionally regulate gene expression by base-pairing with the 3' untranslated region of complementary messenger RNA targets [4,5]. miRNAs were found to be present in human plasma and serum in a remarkably stable and cell independent form, making their potential as novel non-invasive biomarkers for physiological and pathophysiological conditions, including cancer, of growing interest [6,7].

Although circulating exosomes and their content can now serve as biomarkers for various diseases, however, due to limited space and availability of −80 °C freezers, many hospitals and laboratories store patient plasma/serum samples at −20 °C and even at 4 °C, for many years. Therefore, the aim of our study was to investigate the impact of differential storage conditions as well as differential treatment on the stability and abundance of individual and total miRNAs in human plasma and plasma exosomes.

## 2. Results and Discussion

**Figure 1:**
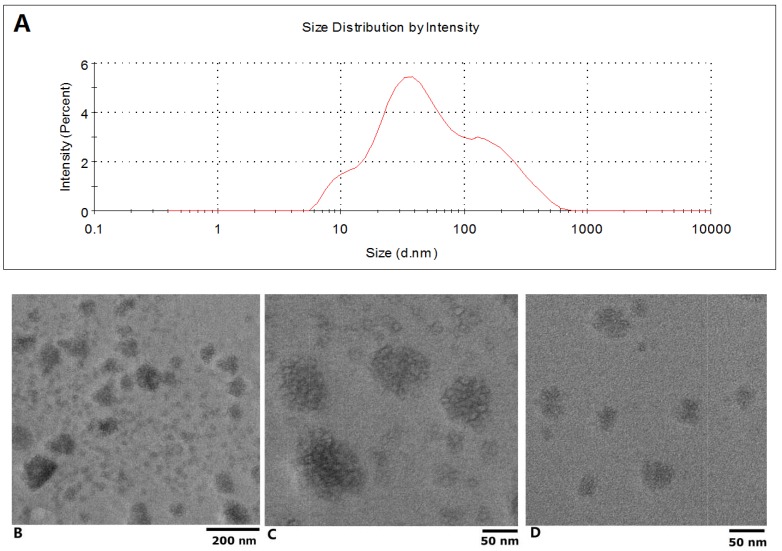
Characterization of the isolated exosome by TEM and Grainsize Analyzer. (**A**) Sizes distribution of the plasma exosome; (**B**) Photograph of plasma exosome with low-magnification TEM; (**C**) and (**D**) High resolution TEM examination results.

The isolated exosomes were characterized by TEM and Grainsize Analyzer first. As can be seen in Figure 1A, a two peak curve was obtained from the isolated exosomes with a zetasizer, the z-average was 48.42 d.nm; the sizes of the two peaks were 42.40 ± 25.98 nm and 126.9 ± 58.3 with weighted intensity of 68.2% and 31.8%, respectively; the polydispersity index was 0.576. The electron microscope images of exosomes are shown in Figure 1. Based on low-magnification TEM images (JEOL JEM-2100, 200 keV), the size of these nanoparticles could be roughly classified into two groups (Figure 1B), those with diameters ranging from about 10 to 50 nm and others with diameters below 100 nm (Figure 1C,D). From the figures, it can be seen that the nanoparticles have a generally spherical shape. Subsequent high resolution TEM examination of the exosomes revealed their morphological and structural features; representative images of each group of nanoparticles are presented in Figure 1B–D.

**Figure 2:**
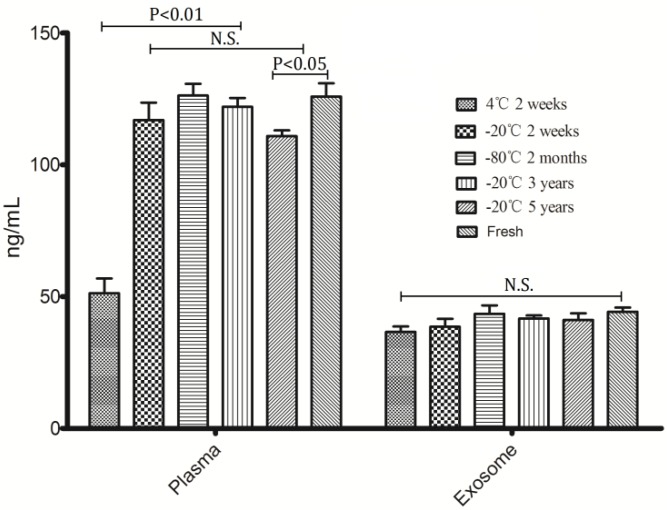
Concentration of RNA extracted from exosomes and plasma under different conditions.

To assess the effect of differential storage temperature and differential storage time on miRNA stability, freshly isolated plasma from healthy volunteers was stored at 4 °C, −20 °C and −80 °C without thawing; exosomes were separated from the stored plasma after 2 weeks, 2 months, 3 years and 5 years. RNAs were isolated from exosome and the corresponding plasma samples. As can be seen from Figure 2, the concentration of exosomal RNA had no significant differences among differential storage conditions when compared with fresh prepared samples; while some differences observed in the corresponding plasma RNA. Plasma RNA at 4 °C was degraded significantly compared with other storage conditions, in addition, long term storage (5 years) at −20 °C also lead to degradation of plasma RNA, the *p*-value is less than 0.05 when comparing with fresh prepared samples and other temperature conditions storage. Due to the fact that −20 °C freezers are available in most institutions and hospitals, storage condition of room temperature (RT) were not investigated in this study.

**Figure 3:**
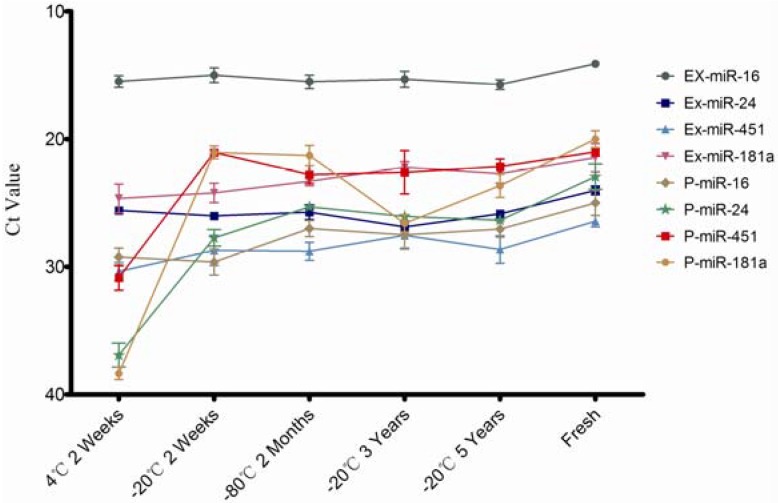
Quantitative results of individual miRNA in plasma and exosomes under different conditions. EX-miR denoted miRNAs from exosomes; P-miR denoted miRNAs from plasma.

The miRNA concentration was measured through individual specific miRNAs (miR-16, miR-24, miR-451, and miR-181a) by qPCR and these miRNA expression levels in exosomes and plasma except exosomes which were simultaneously detected. The results are shown in Figure 3. These four serum/plasma abundant miRNAs are quite stable in exosomes (Figure 3), consistent with the Qubit assay results as shown in Figure 2. As expected, these individual miRNAs expression quantified by qPCR is decreased remarkably when stored at 4 °C, except miR-16, even so, all these miRNAs were still detectable after two weeks at 4 °C. This suggests that plasma samples can stored for short periods at 4 °C for miRNA investigation. This highlights the superior stability of plasma and exosomal miRNAs compared to its mRNA [8].

**Figure 4:**
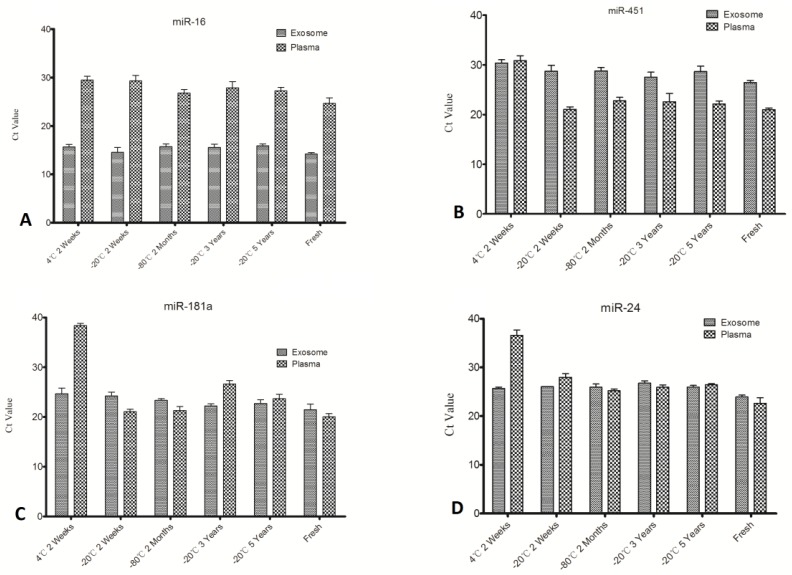
Comparison of individual miRNA level in exosomes and plasma.

No significant expression level differences were obtained except for miR-16 from plasma out of the four selected miRNAs (Figure 3 and Figure 4), while others all degraded greatly under unsuitable storage conditions. This finding suggests differences in stability of individual plasma miRNAs at different time points and temperatures. To further explore this matter, miRNA expression levels of exosomes and plasma were compared (Figure 4). We can see from Figure 4A that expression of miR-16 in exosomes is significantly (*p* < 0.01) higher than plasma, while miR-451 in exosomes is lower when compared with its expression in plasma (Figure 4B). The other two miRNAs assayed in this study showed no significant differences (Figure 4C,D). It is inferred that individual miRNA expression levels in exosomes and outside exosomes is irregular; some miRNA may be contained mainly in exosomes and the rest is outside exosomes.

**Figure 5:**
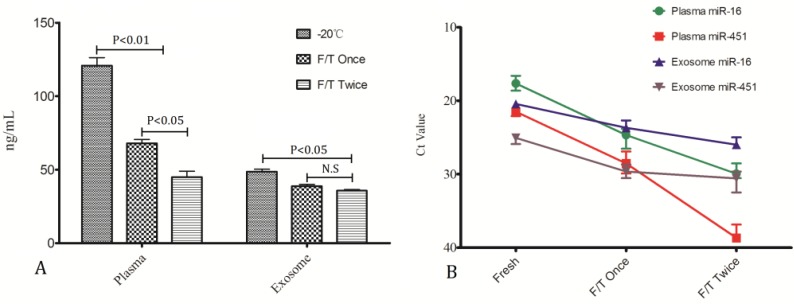
Impact of freeze/thaw cycles on miRNA stability.

To investigate whether the miRNA stability be impacted by freeze/thaw(F/T) cycles, RNA in plasma samples stored at −20 °C with one F/T cycle and two cycles of F/T were compared with samples under continuous storage at −20 °C without thawing. The results can be seen in Figure 5. Plasma RNA concentration was decreased greatly after one cycle of freeze-thawing (*p* < 0.01) and further reduced after two thawing cycles, whereas, exosome RNA tended to be less affected by freeze/thaw cycles (Figure 5A), and differences were detected only after two FT cycles (*p* < 0.05). As can be seen from Figure 5B in individual miRNA measurements similar results were obtained. No significant level differences were found in miR-16 and miR-451 from exosomes while they decreased remarkably in plasma.

## 3. Experimental

### 3.1. Sample Collection

All samples used in this study were collected from eight volunteers who have undergone an annual physical examination in our school infirmary since 2008. Peripheral blood samples were centrifuged at 1,600 *g* for 10 min for plasma collection followed by an additional centrifugation step at 16,000 *g* for 10 min and filtration through 0.2 µm pore size syringe filter. In order to avoid miRNA expression differences due to inter-individual variations, plasma samples were pooled and aliquoted. The same treatments were applied each year.

The samples in 4 °C for 2 weeks as well as in −20 °C for 2 weeks and −80 °C 2 months were studied as short term storage condition; plasma samples in −20 °C for 3 years and 5 years were as long term storage conditions in this study. Freezing and thawing cycles were also considered as specific storage conditions may impact the stability of miRNA in plasma and exosomes.

### 3.2. Exosome Isolation

Exosomal fraction from 1 mL of plasma was isolated by ExoQuick™ Exosome Precipitation Solution according to the manufacturer's recommendations (System Biosciences Inc., Mountain View, CA, USA). Briefly, 1/4 volume of ExoQuick Solution was added to plasma and samples were refrigerated at 4 °C overnight. The mixture was centrifuged at 1,500 *g* for 30 min and supernatant was removed by aspiration. Pelleted fraction was re-suspended in nuclease-free water. The isolated exosome was analyzed by Grainsize Analyzer (Zetasizer Nano ZS, Malvern Instruments, Malvern, UK) firstly. The analysis yields the z-average of the sample, which is intensity weighted mean diameter of the bulk population and the polydispersity index, which is a measure for the width of the size distribution. The measuring range of the zetasizer is from approximately 0.1 nm to 10 µm. The sizes and aggregational states of exosome were examined using a TEM (JEM-2100 equipped with JED-2300T energy dispersive spectrometer (EDS) system, JEOL, Tokyo, Japan).

### 3.3. RNA Extraction

RNA was extracted from plasma and exosomal fractions using Qiagen miRNeasy Mini kit (Qiagen, Hilden, Germany) according to the manufacturer’s instructions, with the final elution volume of 50 μL. The concentration of RNA fraction was quantified using Qubit^®^ RNA HS Assay Kit by Qubit^®^ 2.0 Fluorometer (Life Technologies, Grand Island, NY, USA) according to the manufacturer’s protocol. This method has a detection range of 5–100 ng.

### 3.4. MiRNA Selection

Four individual miRNAs (miR-16, miR-451, miR-181a and miR-24) were selected based on their expression in hematopoietic cells, amplification efficacy [9] and previously related studies [10,11,12]. miR-451 was highly abundant in red blood cells and the most abundant in sequencing data of our previously study, whereas miR-24 is ubiquitously expressed in all hematopoietic lineages. miR-16 was included due to its previously published use as a housekeeping gene in plasma samples [13].

### 3.5. qPCR Analysis

The expression level of matured miRNAs was tested by quantitative real time PCR (qPCR) using SYBR^®^ Premix Ex Taq™ II, Perfect Real Time (TaKaRa, Dalian, China) on an Applied Biosystems 7500 real-time PCR machine (Life Technologies). For reverse transcription (RT) reactions, stem-loop primer was applied for cDNA synthesis according to the report by Mestdagh *et al.* [14]. qPCR was performed according to the manufacturer’s instructions and all samples were analyzed in triplicate. The primers for the four miRNAs reverse transcription and quantification were synthesized and purified by Invitrogen Inc. (Shanghai, China) The DNA sequences of primers are listed in Table 1. Mean absolute CT values and standard deviations are displayed on the graphs. Statistics were performed using Student’s t-test and *p* < 0.05 was considered as significant. GraphpadPrism5 (GraphPad Software Inc., La Jolla, CA, USA) were used for further statistical analyses.

**Table 1:** DNA sequences of primers used.

Name	DNA Sequence (5'-3')
miR-451-RT Primer	GTCGTATCCAGTGCAGGGTCCGAGGTATTCGCACTGGATACGAC*aactca*
miR-451-Forward Primer	GAAACCGTTACCATTACTGAGT
miR-16-RT Primer	GTCGTATCCAGTGCAGGGTCCGAGGTATTCGCACTGGATACGAC*cgccaa*
miR-16-Forward Primer	GTAGCAGCACGTAAATATTGGCG
miR-181a-RT Primer	GTCGTATCCAGTGCAGGGTCCGAGGTATTCGCACTGGATACGAC*actcac*
miR-181a-Forward Primer	GAACATTCAACGCTGTCGGTGAGT
miR-24-RT Primer	GTCGTATCCAGTGCAGGGTCCGAGGTATTCGCACTGGATACGAC*ctgttc*
miR-24- Forward Primer	GTGGCTCAGTTCAGCAGGAACA
Universal Reverse Primer	GTGCAGGGTCCGAGGT

## 4. Conclusions

In this present study, the stability of circulating miRNA from plasma and exosomes was investigated. These results underline the stability of circulating miRNAs exposed to different temperatures and different storage times. In addition, storage at −20 °C barely impacted the overall amount of plasma miRNA for at least 5 years, with only minor changes in the level of individual miRNAs. To our surprise, the impact of short term storage at 4 °C on circulating miRNAs is much less than expected. This study further highlights the potential of exosome miRNAs as biomarkers based on their stability under various storage conditions.
